# Computational Thinking in Life Science Education

**DOI:** 10.1371/journal.pcbi.1003897

**Published:** 2014-11-20

**Authors:** Amir Rubinstein, Benny Chor

**Affiliations:** School of Computer Science, Tel-Aviv University, Tel Aviv, Israel; University of British Columbia, Canada

## Abstract

We join the increasing call to take computational education of life science students a step further, beyond teaching mere programming and employing existing software tools. We describe a new course, focusing on enriching the curriculum of life science students with abstract, algorithmic, and logical thinking, and exposing them to the computational “culture.” The design, structure, and content of our course are influenced by recent efforts in this area, collaborations with life scientists, and our own instructional experience. Specifically, we suggest that an effective course of this nature should: (1) devote time to explicitly reflect upon computational thinking processes, resisting the temptation to drift to purely practical instruction, (2) focus on discrete notions, rather than on continuous ones, and (3) have basic programming as a prerequisite, so students need not be preoccupied with elementary programming issues. We strongly recommend that the mere use of existing bioinformatics tools and packages should not replace hands-on programming. Yet, we suggest that programming will mostly serve as a means to practice computational thinking processes. This paper deals with the challenges and considerations of such computational education for life science students. It also describes a concrete implementation of the course and encourages its use by others.

## Background

The “cultural gap” between biological and computational sciences has become increasingly evident in recent years. Life sciences are going through a dramatic biotechnological revolution, producing huge amounts of data, which is often placed in public databases. The analysis of these data requires nontrivial computational ideas. Life sciences curricula, however, have hardly been altered to reflect this revolution [1]–[3]. Some universities require life science students to take an introductory programming course, while others require a course on bioinformatics tools. These courses tend to focus on practical programming skills or on technical handling of bioinformatics tools. Often, not enough emphasis is put on developing abstract and algorithmic thinking skills in such courses. More advanced computational courses are either inapplicable without appropriate background or narrow down to very specific topics.

This gap presumably starts at the classroom, but it lingers later on. Biology in many institutes and labs is still primarily a descriptive science with little computational approaches being used on a daily basis. Computational approaches in this context are not the mere use of tools, but the integration of computational thinking and algorithms to experiments design; to data generation, integration, and analyses; and to modeling. It is often the case that because of the lack of computational background and relevant training, bench biologists employ computational methods as “black boxes” without a deep understanding of the computational concepts, underlying assumptions, and the limitations of such models. The practice of employing computational methods in biology is usually done in one of two flavors: a somewhat “automatic” use of existing bioinformatics tools by biologists or the application of algorithms to biological data by computer scientists and mathematicians. Both modes may result in a misinterpretation of results and in erroneous conclusion making [4]. Biologists are rarely directly involved in the development of mathematical and computational models. This is mostly due to the complexity of such models and the gaps between the biological and computational cultures.

The majority of biological laboratories would greatly benefit from using computational tools on a daily basis and, consequently, from the presence of an “in-house” expert with a solid computational understanding. Indeed, the need to provide life science students with a wider, deeper computational education, beyond just hands-on skills, is being widely recognized [1]–[7]. However, only a few concrete initiatives have so far been implemented. A notable one is the “integrated science” introductory curriculum [1], breaking down traditional disciplinary barriers, developed in Princeton University by David Botstein and William Bialek. Another initiative, at Harvey Mudd College, is the “"*CS5 green*” course [8], [9]: an introductory computer science (CS) course “designed to give the foundations of computer science in the context of solving real and important problems in the biological sciences.” An international conference dedicated to bioinformatics education, RECOMB-BE, was founded in 2009. General CS education conferences (SIGCSE, iTiCSE) also provide venues for discussions and reports on this topic [7], [10]. The education article type of *PLOS Computational Biology* is a notable resource for practical tutorials and opinions. Online courses, such as *Rosalind* (http://rosalind.info/problems/locations), have started to appear, aiming to attract biologists who want to develop programming skills at their own pace. Several books about computational methods, aimed at biologists, have been published recently [11], [12]. These important initiatives promote the incorporation of quantitative computational skills in biology. Still, their influence on life science undergraduate curricula has been somewhat limited so far.

We join the above-mentioned efforts. We urge such an educational revolution in life sciences and propose a novel, stand-alone, concrete educational building block: a non-introductory course, that aims to expose students to the computational “culture” and focuses on developing computational thinking skills [13], rather than on the mere use of existing bioinformatics tools or programming. The course introduces a diverse range of computational concepts and ideas and demonstrates their applicability to life science. We believe this course constitutes a novel, genuine contribution in the area of educational computational biology.

## Incorporating Computational Thinking in Life Sciences

The course we developed, titled “Computational Approaches for Life Scientists” (http://ca4ls.wikidot.com), is targeted specifically for life science students, both advanced undergraduate and graduate. It is a non-introductory course—basic programming is a pre-requisite (see more details about this choice later). The course's primary goal is:

*To develop students' computational thinking skills by exposing them to the abstract, algorithmic, and logical “culture” of computer science, and familiarizing them with fundamental computational ideas and concepts.*

**Figure 1:**
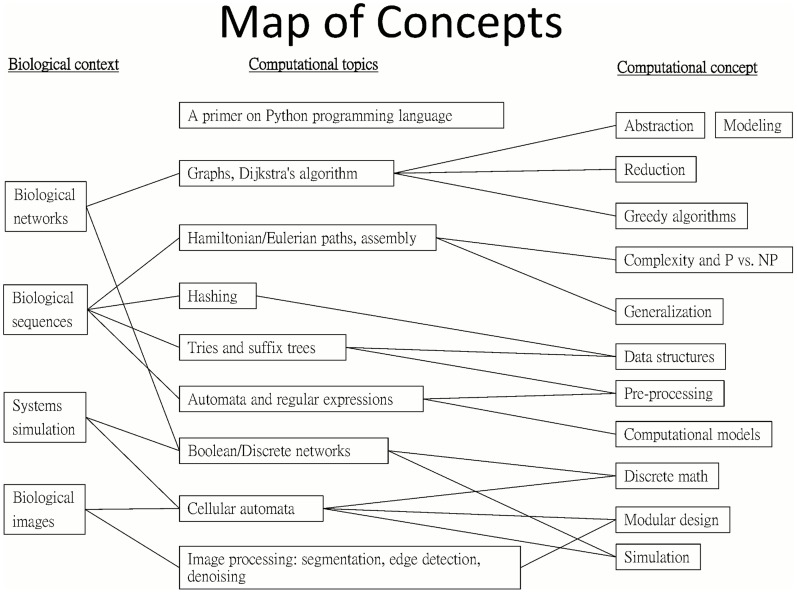
Biological modules of the course and related computational topics.

From the biological point of view, the course consists of four main modules (Figure 1), each corresponding to a different biological domain. We believe it is more accessible to life science students when the course is structured, at high level, in a biologically dominated manner. Each module spans two to four computational topics (one per week) (Figure 1).

The focus of our course is the development of abstract and computational thinking. The design of each module includes four main instructional themes in a “pipeline” structure:

1. Presenting the motivating biological problem and relevant biological background. Given students' biological background, this part is typically rather brief
2. Formulating the problem in computational terms, familiarizing appropriate concepts and notions
3. Dealing with programming issues needed to implement the new ideas
4. Reflecting on the whole process, bringing to light the fundamental computational thinking skills practiced

The supplementary Text S1 presents a detailed example of a “path” through three topics in the pipeline structure. This example is aimed at understanding the principles behind sequence assembly. Figure S1 depicts this process. We remark that here, as well as in other topics in the course, this is a spiral learning process. Students are “walked through” this pipeline more than once, and experience several variants (of increasing complexities) on the computational and biological problems.

Table 1 maps some fundamental computational concepts and thinking processes, and demonstrates topics from our course harnessed to acquire them. We believe that the fourth stage of the suggested instructional pipeline is highly important. Directly naming these concepts, ideas, and processes, discussing them, and reflecting upon them in the context of the new topic will raise students' awareness to them, such that they will be more likely to practice them again in the future.

**Table 1:** Examples for computational concepts and thinking processes discussed in various topics, and emphasized in the “reflection” stage of the pipeline.

Computational concepts and thinking processes	Topics/examples in which they are employed in the course
Abstraction [15]–[17]	Computer representation of biological entities (e.g., graphs for networks, strings for DNA/proteins, matrices of pixels for images)
	Distinction between abstract data types and their implementation (e.g., a graph can be represented as an adjacency matrix or as a neighbors list)
Generalization	From the “bridges of Konigsberg” to conditions for the existence of an Eulerian path in a graph
	From Boolean to discrete models
	From the “Game of Life” to cellular automata
Modular design, decomposition	Image noise reduction and edge detection apply different local morphological operators on image pixels (mean, median, dilation, erosion), thus all are implemented as concrete invocations of a general local operator function
	Simulation of the ”Game of Life” separates GUI, logic (local transition rules) and data control (the “engine” of the simulation)
Reduction [18]	Reducing variants of shortest paths to the shortest path from a single source
	Reducing Hamiltonian path to travelling salesperson, arguing NP-completeness of the former
Pre-processing	Building the suffix tree of a string for later substring matching “Compiling” a regular expression (in Python) for pattern matching
Data structures	Graph
	Stack, used for finding Eulerian paths in a graph
	Priority queue, used for finding shortest paths in a graphs with Dijkstra's algorithm
	Hash table, used as a dictionary, and for the longest common substring problem
	Trie, used as a dictionary for strings
	Suffix tree, used for various string problems
Computational models	Deterministic finite automata (DFA)
	Using DFA for pattern matching
Greedy algorithms	Dijkstra's algorithm
	Regular expressions' evaluation in a greedy manner in Python's re package
Computational complexity; P, NP and NPC	Traveling salesperson and the de novo assembly problem: demonstrating NP-completeness
	Eulerian versus Hamiltonian paths for sequencing by hybridization
	Graph isomorphism
Discrete notions and models	Graphs
	Cellular automata
	Discrete “state graphs” for the simulation of regulation networks

The design of the course was guided by several additional considerations, which we detail below.

### Choice of topics

The course topics span several algorithmic and logical concepts that lie at the heart of CS. These concepts are demonstrated in relevant biological contexts. Two main criteria are considered in the choice of topics: (1) how relevant the topic is for research and practice in life sciences and (2) to what extent the topic can be harnessed to expose students to the computational “culture” and to practice relevant thinking skills. We tackle a wide spectrum of biological and computational issues, appealing to a fairly broad audience among life science students.

### Programming

Even though this is not a programming course, students are required to solve “real-life” biological problems using code. We introduce the programming language Python at the beginning of the course (about two weeks, six hours). It then serves as a vehicle to deliver course topics. While teaching Python, we focus on its practical use, rather than on language syntax and specifications (the latter are more likely to be emphasized in an introductory programming course). Our experience shows that when learning includes concrete, hands-on practice, computational thinking skills are better acquired and underlying concepts are better understood.

### Emphasis on discrete notions

One important choice in the course's design was to exclusively concentrate on discrete approaches such as finite graphs, strings, digital images (represented as a matrix of discrete elements—pixels), finite state automata, etc. These are highly underrepresented in life science curricula, in which continuous notions, such as derivatives, integrals, and differential equations, are taught more widely [12], [14].

### Level of formalism

We choose a level of formalism that matches students' background. Obviously we do not use the same level of formalism as in “pure” CS courses. Nonetheless, we do insist on taking students out of their “cognitive comfort zone” in the sense that we expect them to handle abstract notions and to formalize their statements and algorithms in a rigorous and logical manner. Still, we leave ample time for classroom discussion and for developing intuition and try not to drift into a too-formal or technical instruction.

## Learning Outcomes and Evaluation

Upon successful completion of the course, we expect students to:

- Be familiar with several fundamental concepts and notions in CS, and their applicability to life sciences. Figure 1 lists these computational concepts, and Table 1 describes additional notions related to computational thinking skills
- Be able to identify problems whose manual solution is not feasible, yet they are amenable to a computational solution
- Feel comfortable to communicate with computational biologists/bioinformaticians
- Be able to implement basic solutions to simple biological problems they encounter, and to effectively communicate with more experienced programmers for more complex problems

The course was taught for the first two times in 2013 and 2014 at the Technion, Israel Institute of Technology, Faculty of Biology. In the first round of the course, it was taken for credit by five graduate level and three undergraduate level students. In the second round, it was taken by eight graduate level and nine undergraduate level students. All had elementary programming background in either C, Matlab, or Pascal (a programming course is mandatory for all Technion undergraduate students). Participants were required to submit five home assignments, each including programming tasks and theoretical questions. In the first round, a take-home exam was given at the end, which was replaced in the second round by a final research project: students chose topics that they found interesting among the course subjects, extended them in some manner, and applied them to real biological data. Additional details regarding the projects, and specific project examples, appear in the supplementary Text S2. At the end of the semester, students were either interviewed by the lecturer or asked to fill a survey for feedback. These feedbacks are summarized in the supplementary Text S3.

**Figure 2:**
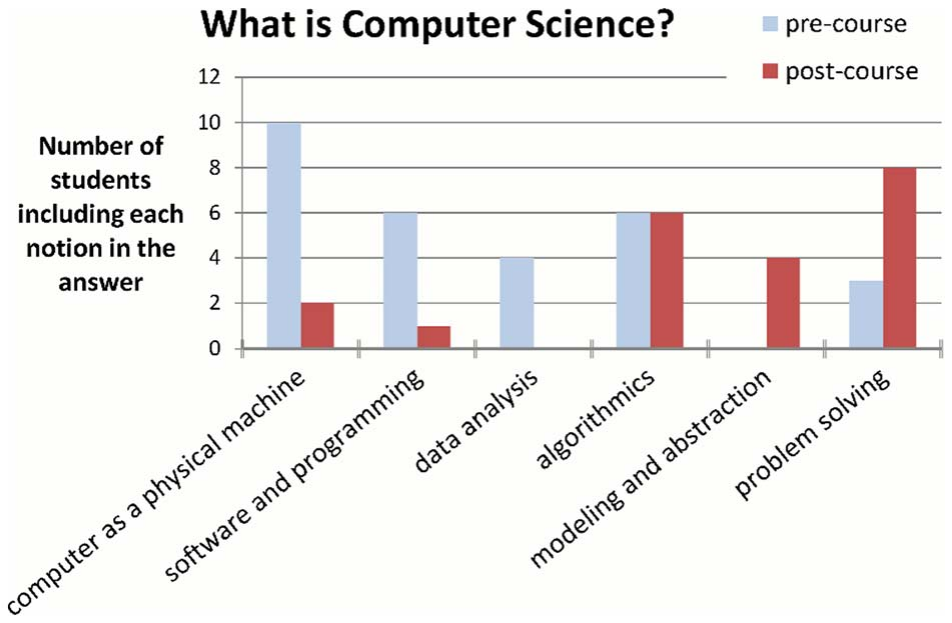
Students' views of the important facets of CS before and after the course. Numbers indicate how many students among the responders included the notion in their definition for the discipline.

To examine the effect of the course on how students view computer science, they were asked to define this discipline before and after the course. Prior to the course, students related the field mostly to the computer as a machine and to software and tools. At the end of the course, however, they tended to relate CS to broader and more abstract terms, such as problem solving and modeling (see Figure 2). We believe this shift in the view of the discipline, especially considering the prior exposure of our students to programming, strengthens the rationale for such a course.

## Discussion

Obviously, there is more than a single way to expose life sciences students to computational thinking. Yet, based on our experience, and on numerous discussions with life scientists and bioinformaticians, we feel that a single one-semester course, which does not assume a basic programming course as a prerequisite, is likely to miss the goal of teaching computational thinking and computational concepts to life science students. If basic programming is taught from scratch, not enough time will be left for the higher level computational concepts and their relations to biology, so the depth of coverage of computational thinking will be smaller. Alternatively, the use of packages could dominate the hands-on experience, “masking” the computational ideas. On the other hand, having such a basic programming prerequisite, as in our course, enables us to take the students a step further, beyond programming and tool handling. This facilitates exploring abstract computational notions, experimenting “first hand” with coding them, and applying the code to concrete biological tasks. We believe that these days, a basic programming course is a crucial component of every science curricula. This leads to the recommendation that basic programming should be taught separately, prior to a computational thinking course. Such a prerequisite will allow the students to digest programming issues well before, so they need not be preoccupied with technical issues while taking a computational thinking course. Furthermore, we feel that the understanding of computational thinking and the “language” of CS are hard to obtain independently. While a student “speaking” this language can easily educate him/herself in the use of bioinformatics tools, the other direction is far less amenable to self-study. Teachers engaged with computational education for biologists are sometimes tempted to make their course as practical as they can (and many students feel more comfortable staying away from abstract topics). While practical skills are, of course, important and motivating, we believe that time and educational effort must be spent on abstract notions and thinking processes: naming, discussing, and reflecting upon them.

Most of these conclusions are supported by the surveys and interviews conducted among course students during the two semesters it was taught. Clearly, a more in-depth evaluation of the course, based on a larger number of participants, is called for. This is planned to take place in future offerings of the course.

In our view, an essential part of any course aiming to teach computational thinking to life scientists is the interaction in class, with an able instructor who is knowledgeable in both computer and life sciences. Class interactions in the form of discussions, guided solutions to problems, naming of thinking processes, and exposure of students to alternative (including incorrect) approaches are at the heart of the learning process in this course. Our four-step pipeline instruction model prevents spending too much time on technical aspects since part of the time is explicitly dedicated to reflection and discussion in class.

We strongly believe that we have an important message to deliver. We propose a way to take life scientists' computational education a step further. Even small steps in this direction are likely to have substantial consequences in life or medical science practices and research in the long run. Such initiatives can greatly contribute to narrowing the gaps between life sciences and bioinformatics/computational biology and motivate other scientists and science education experts to be involved in similar efforts.

## Supporting Information

Figure S1 A “path” through three topics in the pipeline structure.
(TIF)

Figure S2 (A) A microscope slide containing Bacilli anthracis cells and spores (image taken from [2]). (B) Endospores identified (white spots in the original image). (C) Vegetative cells identified (dark spots in the original image).
(TIF)

Figure S3 Students' attitudes towards home assignments difficulty and effectiveness.
(TIF)

Text S1 A path through three topics in the pipeline structure of the course.
(DOCX)

Text S2 Examples for end of course projects.
(DOCX)

Text S3 Feedback from course students.
(DOCX)
