# Supplementary figures and images for: Computational Thinking in Life Science Education
Source: PLoS Comput Biol. 2014 Nov 20;10(11):e1003897. doi: 10.1371/journal.pcbi.1003897 (PMC4238948; doi:10.1371/journal.pcbi.1003897)

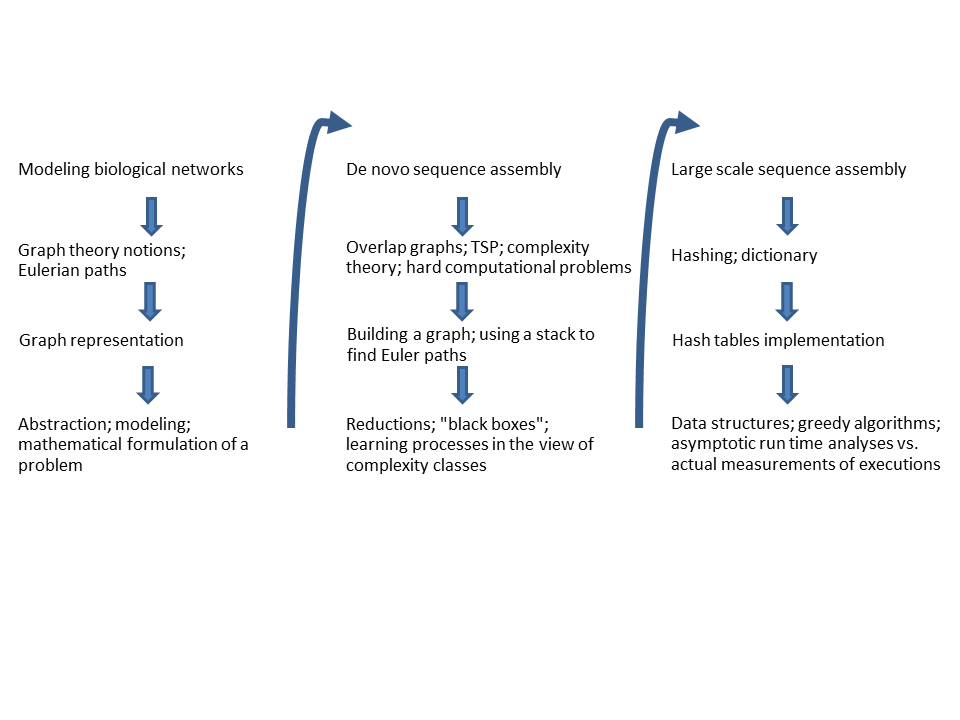

Supplement: Figure S1 — A “path” through three topics in the pipeline structure. (TIF) [file pcbi.1003897.s001.tif]

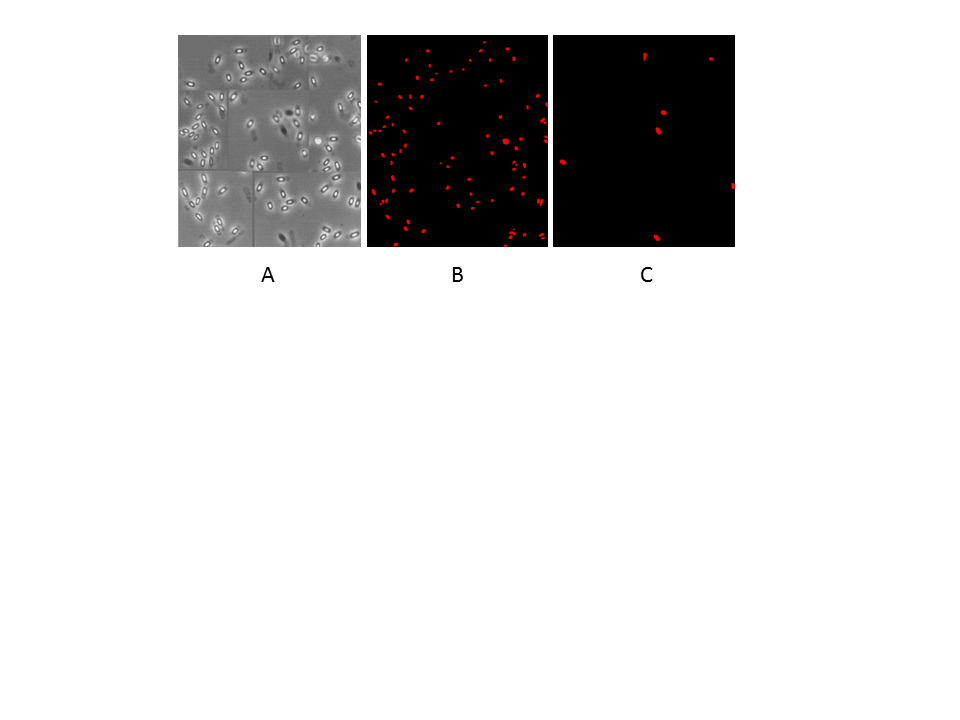

Supplement: Figure S2 — (A) A microscope slide containing Bacilli anthracis cells and spores (image taken from [2]). (B) Endospores identified (white spots in the original image). (C) Vegetative cells identified (dark spots in the original image). (TIF) [file pcbi.1003897.s002.tif]

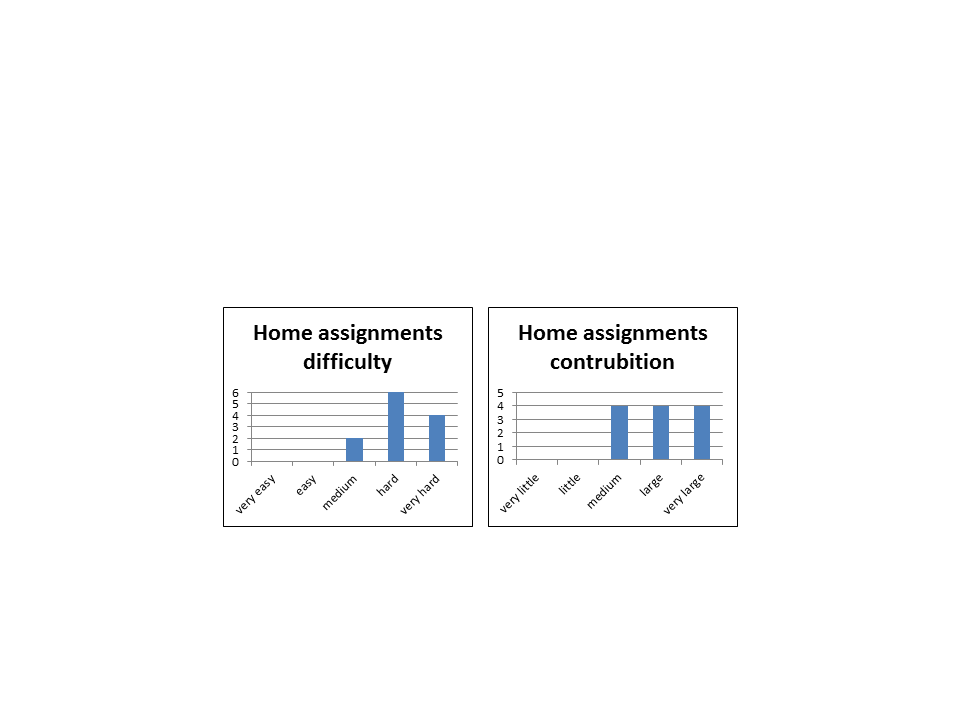

Supplement: Figure S3 — Students' attitudes towards home assignments difficulty and effectiveness. (TIF) [file pcbi.1003897.s003.tif]
