# Supplementary material for: Computational Thinking in Life Science Education
Source: PLoS Comput Biol. 2014 Nov 20;10(11):e1003897. doi: 10.1371/journal.pcbi.1003897 (PMC4238948; doi:10.1371/journal.pcbi.1003897)
Supplement: Text S1 — A path through three topics in the pipeline structure of the course. (DOCX) [file pcbi.1003897.s004.docx]

**Supplementary 1: A path through three topics in the pipeline structure of the course**

We describe a specific “path” through three topics in the pipeline structure, aimed at understanding the principles behind sequence assembly (see Fig. S1). The computational "ingredients" include graph theory, computational complexity, and hashing. Biologists reading these paragraphs are possibly not familiar with some of these topics, yet the following description intends to give the intuition for their relevance in the biological context. We remark that here, as well as in other topics in the course, the learning process is spiral: students are "walked through" this pipeline more than once, and experience several variants (of increasing complexities) on the computational and biological problems.

The first iteration starts with a short introduction to networks, and their applicability in various biological settings (such as protein-protein interactions, regulation, metabolism, etc.). In order to represent and analyze such networks, basic graph theory notions are introduced, and some mathematical formulation (*e.g*., of various graph properties) is described and practiced. Next, we present the relatively simple notion of Eulerian paths (a path in a graph is called Eulerian if it visits every edge exactly once). At this point, the focus is on the conditions for the existence of such paths in a graph, not on actually finding them. We then move on to programming: introducing different ways to represent graphs in the computer, and implementing (in Python) a function that detects the existence of Eulerian paths or lack thereof. We conclude this first iteration with a reflection on the concepts of modeling and abstraction, and the importance of expressing statements in a formal manner.

The second iteration starts with de novo sequence assembly, a contemporary topic in new generation sequencing. The problem is formulated as finding a shortest superstring that contains a set of DNA reads (at this point, it is assumed, for simplicity, that there are no read errors). We explain that this is a form of a one dimensional jigsaw puzzle, and then the notion of overlap graphs (in which nodes represent reads, and edges represent overlaps between reads) is introduced. We explain how this problem can be viewed as the (famous) travelling salesperson problem (TSP). Next, we provide a brief introduction to complexity theory: the complexity classes P, NP and NPC, and the notion of computationally hard problems are explained at a high level, including examples such as Hamiltonian paths, graph isomorphism, etc. Students are now able to realize that the TSP does not provide a feasible solution to the assembly problem. This leads to a revised formulation of the problem, using the terminology of sequencing by hybridization. This new formulation changes our problem back to finding Eulerian paths. At this point we introduce the efficient algorithm for finding Eulerian paths. The programming in this stage includes identifying all overlaps among pairs of reads, building the edge-overlap graph, and using a data structure called stack to find Eulerian paths. This iteration ends with an intuitive introduction of the notion of reduction: we show how reductions help us order problems by levels of difficulty^[[1]](#footnote-1)^, and emphasize the notion of black-boxes (and abstraction, again) in that context. To recap, we discuss how cognitive learning processes can be viewed as reducing a problem’s difficulty level.

In the third iteration, we take sequence assembly a step further: Although theoretically efficient, the naïve approach for finding overlaps between reads turns out inefficient when the number of reads is large (order of millions, which in reality is usually the case). Therefore, we take a different approach and introduce the notion of hashing. The abstract “dictionary” data structure is defined, and hash tables are introduced to efficiently implement it. We use Python’s built-in hash function to implement and execute assembly on synthetic, as well as real datasets^[[2]](#footnote-2)^. For that, we develop a greedy algorithm, which tries to expand existing reads into longer fragments as much as possible. At the end of this part, we recap on theoretic complexity analysis vs. "real" time performance. We also discuss how simplifying assumptions are often made to enable an efficient enough solution; in our case, for example, assuming there are no long repeats in the underlying “genome”, and no errors in the reads. By now, students have seen several data structures in the course (stack, queue, hash table, trie, etc.), and this is a good point to make some high level comparison between them. A brief discussion of greedy algorithms and their pitfalls concludes this part.

1. At a later stage, after teaching Dijkstra’s algorithm for shortest paths in a graph, we also show reductions in a “positive” context: using existing solutions in order to solve new problems. [↑](#footnote-ref-1)
2. In other parts of the course we show additional applications of hashing, *e*.*g*., finding a longest common subsequence of two proteomes. [↑](#footnote-ref-2)
