# Supplementary material for: Computational Thinking in Life Science Education
Source: PLoS Comput Biol. 2014 Nov 20;10(11):e1003897. doi: 10.1371/journal.pcbi.1003897 (PMC4238948; doi:10.1371/journal.pcbi.1003897)
Supplement: Text S2 — Examples for end of course projects. (DOCX) [file pcbi.1003897.s005.docx]

**Supplementary 2: Examples for end of course projects**

At the end of the course, each student or pair of students was required to work on a research project, based on computational approaches studied in the course. The goals of the projects were: (1) to practice the application of computational approaches in order to solve a real biological problem, and (2) to expose students to the process of academic research, including academic writing. The projects had to combine three ingredients: biological – defining a real biological problem; computational – designing a solution; programming - implementing the solution in Python. In accordance with the second goal, the project report had to be structured as a short academic paper, including an abstract, introduction, methods, results and discussion sections. Following are three examples of students' projects. The full reports appear at the course website.

Example 1: **A simple discrete model of extrinsic and intrinsic apoptosis pathways in mammalian cells**. The student used and enhanced a discrete model for regulation networks, taught in the course, for the simulation of the apoptosis pathway. This model, as well as other Boolean and discrete models, has been shown to be effective in elucidating various biological systems, especially when detailed quantitative data is missing, or when continuous methods are computationally intractable. For details about the model, see [1] or the course website at [www.ca4ls.wikidot.com](http://www.ca4ls.wikidot.com). The student built a simplified representation of part of the apoptosis network, which exhibited the expected initiation of the apoptosis pathway. The student then showed how triggering both extrinsic (death receptor) and intrinsic **(**mitochondrial**)** pathways of apoptosis amplifies the level of the caspase-3 protein, in agreement with the literature.

Example 2: **Quantification of bacteria cells, using image processing techniques and interactive GUI**. The student used image processing techniques taught in the course, in order to evaluate the stage of germination in a spore forming bacteria (for example *bacillus* or *clostridium*). The challenge was to distinguish between two stages in the developmental process: endospores and vegetative cells (see Fig. S2 and [2]). The student used the techniques of image segmentation to distinguish between different cell types, and image erosion to remove the halo around spores, facilitating the segmentation. The existing Python packages SciPy was also used to count the number of bacteria cells in the resulting images. The implementation was incorporated into a GUI environment previously used in the course, allowing the user to simply click a button and get a count of the two types of cells in a given image.

Example 3: **k-mer based DNA analysis**. This project tackled a basic challenge of the metagenomic approach in microbial ecology: the assignment of genomic fragments to a certain species or taxonomic group, when suitable marker genes are absent. The students hypothesized, based on previous work, that DNA analysis based on varying k-mer lengths will provide finer details as to the taxonomic classification of the organisms in a given data set, when compared to similar analysis based on k=1 (which is equivalent to the GC-content of the DNA). They analyzed a set of over 80 PCR DNA amplicons of a photosynthetic gene, amplified from a single drop of water. Their analysis included a visualization of the results using a SciPy clustering module and Matplotlib package for generating heat maps.


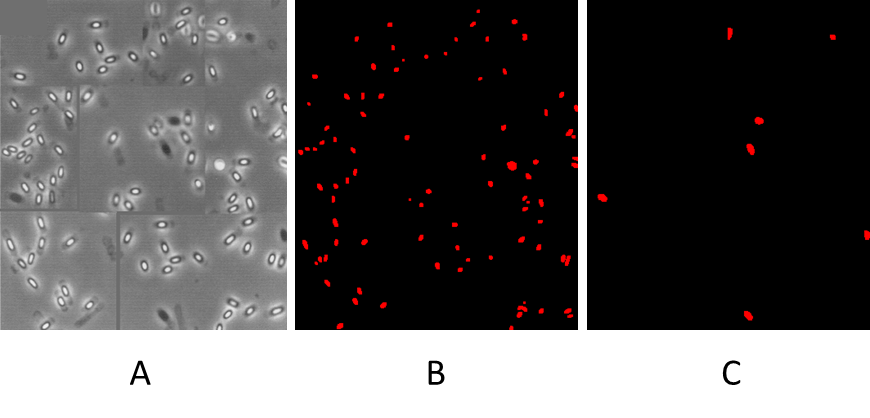


**Figure S2: (A) A microscope slide containing Bacilli anthracis cells and spores (image taken from [2]). (B) Endospores identified (white spots in the original image). (C) Vegetative cells identified (dark spots in the original image).**

**References:**

1. Amir Rubinstein, Ofir Hazan, Benny Chor, Ron Y Pinter and Yona Kassir, "The effective application of a discrete transition model to explore cell-cycle regulation in yeast", BMC Research Notes 2013, 6:311.
2. Nicholson, W.L. and B. Galeano, *UV resistance of Bacillus anthracis spores revisited: validation of Bacillus subtilis spores as UV surrogates for spores of B. anthracis Sterne.* Appl Environ Microbiol, 2003. 69(2): p. 1327-30.
