# Supplementary material for: Computational Thinking in Life Science Education
Source: PLoS Comput Biol. 2014 Nov 20;10(11):e1003897. doi: 10.1371/journal.pcbi.1003897 (PMC4238948; doi:10.1371/journal.pcbi.1003897)
Supplement: Text S3 — Feedback from course students. (DOCX) [file pcbi.1003897.s006.docx]

**Supplementary 3: Feedback from course students**

Almost all students mentioned Python as being simple to use and flexible. Preferred topics were fairly diverse, yet image processing was the most popular. All students said that the course was very different from “standard” programming courses, which focus on programming language issues and are much less relevant to life sciences. Students that previously took a bioinformatics tools course mentioned that it taught them how to solve more restrictive types of problems, in a less independent manner. Several students commented that the P vs. NP topic, although very theoretical, contributed to their awareness of the types of limitations in computer science. Most students preferred more hands-on experience in the course, while only one preferred more theoretical content. On average, students said that the homework assignments were difficult, yet helpful for understanding the course topics (see Fig. S3). Students were asked whether at the end of the course they have a better understanding of the “culture” and way of thinking in computational biology and computer science. On a scale of 1 (totally disagree) to 5 (totally agree), 9 out of 11 responders marked 5 (the other two marked 4).
